# Natural Prevalence, Molecular Characteristics, and Biological Activity of *Metarhizium rileyi* (Farlow) Isolated from *Spodoptera frugiperda* (J. E. Smith) Larvae in Mexico

**DOI:** 10.3390/jof10060416

**Published:** 2024-06-08

**Authors:** Yordanys Ramos, Samuel Pineda-Guillermo, Patricia Tamez-Guerra, Alonso Alberto Orozco-Flores, José Isaac Figueroa de la Rosa, Selene Ramos-Ortiz, Juan Manuel Chavarrieta-Yáñez, Ana Mabel Martínez-Castillo

**Affiliations:** 1 Instituto de Investigaciones Agropecuarias y Forestales, Universidad Michoacana de San Nicolás de Hidalgo, Km. 9.5 Carretera Morelia-Zinapécuaro, Tarímbaro 58880, Michoacán, Mexico; yordanys.ramos@umich.mx (Y.R.); jose.figueroa@umich.mx (J.I.F.d.l.R.); manuel.chavarrieta@umich.mx (J.M.C.-Y.); 2 Facultad de Ciencias Biológicas, Universidad Autónoma de Nuevo León, Av. Pedro de Alba s/n, Ciudad Universitaria, San Nicolás de Los Garza 66455, Nuevo León, Mexico; patamez@hotmail.com (P.T.-G.); lacxelo@gmail.com (A.A.O.-F.); 3 Consejo Nacional de Humanidades, Ciencias y Tecnologías (CONAHCYT)—Instituto de Investigaciones Agropecuarias y Forestales, Universidad Michoacana de San Nicolás de Hidalgo, Km. 9.5 Carretera, Morelia-Zinapécuaro, Tarímbaro 58880, Michoacán, Mexico; selene.ramos@umich.mx

**Keywords:** biocontrol, entomopathogenic fungi, fall armyworm, Noctuidae, pathogenicity, virulence

## Abstract

Entomopathogenic fungi have been considered potential biological control agents against the fall armyworm *Spodoptera frugiperda* (J. E. Smith), the world’s most important pest of maize. In this study, we evaluated the natural infection, molecular characteristics, and biological activity of *Metarhizium rileyi* (Farlow) isolated from *S. frugiperda* larvae of this insect, collected from maize crops in five Mexican locations. Natural infection ranged from 23% to 90% across all locations analyzed. Twenty-four isolates were evaluated on *S. frugiperda* second instars at a concentration of 1.0 × 10^8^ conidia/mL, causing 70% to 98.7% mortality and 60.5% to 98.7% sporulation. Isolates T9-21, Z30-21, PP48-21, and L8-22 were selected to determine their phylogenetic relationships by *β-tubulin* gene analysis and to compare median lethal concentration (CL_50_), median lethal time (LT_50_), and larval survival. These isolates were grouped into three clades. The T9-21, PP48-21, and J10-22 isolates were closely related (clade A), but phylogenetically distant from Z30-21 (clade B) and L8-22 (clade C) isolates. These genetic differences were not always reflected in their pathogenicity characteristics since no differences were observed among the LC_50_ values. Furthermore, isolates T9-21, J10-22, and L8-22 were the fastest to kill *S. frugiperda* larvae, causing lower survival rates. We conclude that native *M. rileyi* isolates represent an important alternative for the biocontrol of *S. frugiperda*.

## 1. Introduction

Maize (*Zea mays* L.) is one of the three most consumed cereals worldwide, alongside rice (*Oryza sativa* L.) and wheat (*Triticum aestivum* L.) [1]. This cereal serves as a significant food source for both humans and livestock due to its high protein content [2]. Mexico is the seventh largest maize producer globally, with an annual production of over 21 million tons on more than six million hectares of cultivated land [3]. The fall armyworm, *Spodoptera frugiperda* (J. E. Smith) (Lepidoptera: Noctuidae) is one of the main limiting factors of maize production. This insect is native to the Americas [4], but since 2016, it has rapidly spread and is now considered to be a major invasive pest in Africa, Asia, and Australia [5]. The larval stages mainly feed on the developing leaves of maize plants, thereby limiting the crop’s photosynthetic potential and reducing the overall yield by up to 58% [6].

Control of this pest is mainly achieved through the application of broad-spectrum insecticides at frequent intervals. However, this control strategy is not completely successful because of the insect’s potential to develop resistance toward the majority of conventional chemical ingredients [7]. In addition, these chemicals may cause negative side effects on human health and the environment, reducing beneficial arthropod populations, and increasing crop production costs [8]. Therefore, there is a recognized need to find alternatives for pest control that are compatible with integrated pest management (IPM) practices [9]. Thus, biological control, through the use of entomopathogenic fungi, has attracted particular interest [10,11].

Entomopathogenic fungi, a special group of soil-dwelling microorganisms, are a valuable tool that has been successfully implemented against many species of agricultural pests [12,13]. These entomopathogens infect all life stages of their hosts. They are well adapted to target insects’ habitats and have lower risks of affecting non-target organisms within the agroecosystems [14]. Species of entomopathogenic fungi from the *Beauveria* (Vuillemin) and *Metarhizium* (Sorokin) genera are among the most common and effective control agents used as biological insecticides [15,16]. *Metarhizium rileyi* (Farlow) Kepler, SA Rehner, and Humber (formerly *Nomurea rileyi*) (Hypocreales: Clavicipitaceae) is a cosmopolitan pathogen that infects many species of Lepidoptera [10,17,18]. Natural epizootics of this fungus have been reported in several noctuid pests [19,20,21,22,23], including *S. frugiperda* [24,25,26,27,28]. Although *M. rileyi* has been recognized as an effective control agent to complement integrated pest management strategies [10], its efficacy in causing mortality is isolate-specific [5]. Therefore, it is important to identify and characterize native isolates of this fungus that cause natural epizootics in *S. frugiperda* populations. In this study, we investigated the natural infection of *M. rileyi* isolated from *S. frugiperda* larvae collected in five maize-producing locations in Mexico and compared the biological activity and sporulation rate of 24 isolates in second instars of this pest. We then selected five isolates to determine their phylogenetic relationships using the *β-tubulin* gen [18], which can resolve higher-level taxonomic relationships [29], and to compare their biological activity related to median lethal concentration (LC_50_), median lethal time (LT_50_), and larval survival. In this study, we refer to pathogenicity as the pathogen’s capacity to enter the host, establish infection, reproduce, and cause death (as measured in concentration–mortality metrics), and virulence as the median time elapsing between inoculation and host death [30].

## 2. Materials and Methods

Unless different conditions are specifically detailed below, fungal growth (polysporic and monosporic cultures), microcultures, conidial viability, and bioassays were evaluated in the laboratory at 25 °C ± 2 °C, 75% ± 5% relative humidity (RH) in darkness.

### 2.1. Insect Rearing

Insects used in this study were obtained from an *S. frugiperda* colony maintained in the Laboratorio de Entomología Agrícola of the Instituto de Investigaciones Agropecuarias y Forestales (IIAF), Universidad Michoacana de San Nicolás de Hidalgo (UMSNH) in El Trébol, Tarímbaro, Michoacán State, Mexico. Larvae were individually reared in 30 mL plastic cups with a piece of semi-synthetic diet [31], without formaldehyde. Pupae were confined in 0.5 L plastic containers and, after emergence, adults were transferred to brown paper bags (18 × 11 × 40 cm) in an approximate ratio of 1:1 (male: female). Adults were fed with a 15% honey solution. The paper bags were daily replaced once egg laying had started. The whole rearing process was performed in an environmental chamber at 25 °C ± 2 °C, 70% ± 5% RH, and a photoperiod of 16:8 h (light–darkness).

### 2.2. Fungal Isolates

We collected 295 *S. frugiperda* larvae from maize crops in five locations in the municipalities of Tarímbaro, Zinapécuaro, Pátzcuaro, and Chucándiro in Michoacán, Mexico, during the rainy season in September 2021 and 2022 (Table 1). This region has a mean maximum day-time temperature of 28 °C (ranging from 27 °C to 29 °C) and a minimum night-time temperature of 9 °C (ranging from 9 °C to 10 °C), with a mean RH of 79% (ranging from 76% to 84%) in September (data obtained from www.meteored.mx, accessed on 1 March 2024).

Larvae were directly collected from maize plants by walking through the field. Larval collections were made at different phenological stages of maize plants (from V10 to V15 [32]. Following collection, larvae were placed in plastic containers with maize leaves and transported to the Laboratorio de Patología de Insectos-IIAF-UMSNH. The geographical coordinates were recorded at each collection site using a GPS device.

In the laboratory, larvae were individually placed in 30 mL plastic cups containing a semi-synthetic diet and daily monitored to observe evidence of fungal infection or the presence of other pathogens until pupation. Eighty-nine fungal isolates were obtained, each corresponding to one infected larva (as detailed in the Section 3). If larvae displayed signs of mycosis, polysporic cultures were performed. For this, conidia were taken from the infected larva using a sterile microbiological loop and then inoculated onto maltose peptone agar medium fortified with yeast extract (MPYA: 40 g/L maltose, 10 g/L casein peptone, 10 g/L yeast extract, and 15 g/L agar), containing 0.05% streptomycin sulfate and 0.04% chloramphenicol [33]. All fungal cultures were incubated for 14 days.

Microcultures were then performed to determine conidial viability. For this, a sample of conidia from each polysporic culture was obtained and deposited in 1.0 mL of a 0.05% Tween 80 sterile solution. Afterward, a 100 μL volume of this suspension was taken and adjusted at a concentration of 1 × 10^8^ conidia/mL. This sample was placed on 250 μL of solidified MPYA medium on a standard sterilized microscope slide. The number of germinated conidia was counted at 24 h after incubation. Conidial germination was considered to have occurred when the germ tube length was at least two times the length of the conidia [34]. The percentage of conidial germination was calculated using the following formula: % conidial germination = (V/T) × 100, where V is the total number of viable conidia, and T is the total number of viable and non-viable conidia in the sample [35]. Fungal isolates were considered viable if their conidial germination was ≥95% [36,37].

Morphological identification of all fungal isolates was performed using the keys proposed by Humber [38] under an optical microscope at 400× magnification.

Of the 89 isolates mentioned above, 24 were selected to determine their biological activity. The selection was based on isolates that had between 95% and 100% conidia viability. Monosporic cultures were obtained from these 24 isolates as described by Goettel and Inglis [34], with the exception that the conidia were incubated at 25 ± 2 °C instead of 28 °C. Next, conidia were scraped off using a sterile spatula, following the method described by Ramakuwela et al. [39], and suspended in 100 mL of sterile distilled water.

### 2.3. Single-Concentration Bioassays

A group of 20 second-instar *S. frugiperda* (8 h to 10 h after molting) was dipped for 20 s in 3 mL of conidia suspension comprising 1 × 10^8^ conidia/mL for each of the 24 isolates, according to Goettel and Inlgis [34]. Conidial quantification was performed in a Neubauer^®^ chamber under optical microscopy at 400×, using 0.05% (*w*/*v*) Tween 80 to improve conidia dispersion. After treatment, larvae were individually placed in ventilated 30 mL plastic cups, with a piece of semi-synthetic diet. After 24 h, larvae were individually transferred into 2 cm^2^ cylindrical wells of 24-well tissue culture plates containing a semi-synthetic diet. Each group of 20 larvae was considered as one replicate. Four replicates per isolate were prepared. For the control group, larvae were dipped in sterile distilled water with 0.05% Tween 80 solution alone. Bioassays were observed every 24 h to determine the number of dead larvae for each isolate for a 13 day-period. Once mortality was observed, larvae were individually placed into sterile individual wells of a 24-well tissue culture plate containing filter paper with 500 µL of sterile distilled water to promote sporulation. The proportion of cadavers presenting sporulation was then registered.

Based on the bioassay results and the collection sites of the 24 isolates, we selected five isolates (named T9-21, Z30-21, PP48-21, J10-22, and L8-22), which were collected from different sampling sites (Table 1) to examine their genetic characteristics using the *β-tubulin* gene sequences and biological activity.

### 2.4. Genetic Characterization

#### 2.4.1. DNA Extraction

DNA extraction: Mycelium from T9-21, Z30-21, PP48-21, J10-22, and L8-22 isolates was individually obtained by inoculating 100 mL of Sabouraud dextrose broth (SDB) (Bioxon, Mexico City, Mexico) culture medium with a 10 mm plug taken from 20-day-old fungal cultures grown on MPLA. The inoculated SDB medium was incubated for seven days at 25 °C and 120 rpm on an orbital shaker in darkness. Mycelium was then harvested by filtration [40], washed twice with sterile water, and stored at −70 °C until use. Fungal mycelium (30 mg for each isolate) was added to 2 mL microtubes and treated with cetyl trimethyl ammonium bromide (CTAB) extraction buffer for 5 min, previously heated to 65 °C. This mixture was triturated using a micropestle and incubated at room temperature for 5 min. Next, a 600 µL volume of phenol–chloroform–isoamyl alcohol (25:24:1) was added and mixed for one minute. This mixture was centrifuged at 14,000 rpm for 12 min at 8 °C. The supernatant (400 µL) was transferred to new microtubes, and 100 µL of 10 M sodium acetate was added. Microtubes were inverted, and 500 µL of isopropanol was added. The mixture was stored for 24 h at −20 °C to facilitate DNA precipitation, after which the sample was centrifuged at 14,000 rpm at 8 °C for 5 min. The isopropanol was decanted, and one milliliter of cold 70% ethanol was added, mixed, and incubated for 5 min at room temperature, after which it was centrifuged again under the same conditions described above. Next, ethanol was decanted, and the pellet was air-dried for 1 h. Finally, the pellet was suspended in 50 µL of water and the concentration of DNA was determined in a NanoDrop Lite (Thermo Fisher Scientific Inc., Waltham, MA, USA).

#### 2.4.2. Amplification of the β-Tubulin Gene

The amplification of the *β-tubulin* gene was performed by the polymerase chain reaction (PCR) with the primers TUB-F (5′-TGG GCY AAR GGY CAC TAC ACY GA-3′) and TUB-R (5′-TCA GTG AAC TCC ATC TCR TCC AT-3′). Amplifications were performed in a final volume of 50 µL, containing 25 µL of Go Taq^®^ Green Master Mix (Promega, Madison, WI, USA), 1.25 µL of each primer, and 10 ng of genomic DNA. Cycling conditions consisted of an initial denaturation of 2 min at 95 °C followed by 10 s at 95 °C, 30 s at 50 °C, one minute at 72 °C, 34 cycles for 10 s at 95 °C, and a final extension for 5 min at 72 °C. PCR products were separated on a 1.0% agarose gel using SB buffer (1 M boric acid, 0.25 M sodium hydroxide, pH 8.5). Bands were then visualized by staining with ethidium bromide (Thermo Fisher Scientific, Waltham, MA, USA) and captured using the GelDoc EQ gel imaging system (Bio-Rad Laboratories, Hercules, CA, USA), with Image Lab Software ver. 5.2.1 (Bio-Rad Laboratories, CA, USA). Bands were then compared with a 100 bp DNA ladder (Jena Bioscience, Jena, Germany) for scoring. PCR products were purified using the Wizard SV gel and PCR clean-up system kit (Promega), after which Sanger bidirectional sequencing was performed by the National Laboratory of Genomics for Biodiversity (LANGEBIO, Irapuato, Mexico), using the primers TUB-R/TUB-F. The forward and reverse sequences were assembled and edited to obtain a consensus sequence, using the Geneious software version 2013.1.2 (Biomatters, Auckland, New Zealand).

The sequences of the five isolates were submitted to the GenBank public collection of the National Center for Biotechnology Information (NCBI) under consecutive accession numbers from OR589405 to OR589409. The consensus sequences were compared with the *β tubulin* sequences from the databases, using the Basic Local Alignment Search Tool (BLAST version 2.15.0) from the NCBI. Sequences with the highest similarity were downloaded in FASTA format to be used as references in the phylogenetic analyses. The phylogenetic analysis was performed using MEGA 11 version 11.0.13, using the neighbor-joining tree method, with 1000 bootstrap replicates. Phylogenetic distances were computed using the maximum likelihood composite method based on the number of substitutions per site, excluding all positions with gaps and missing data.

### 2.5. Conidia Concentration–Mortality and Speed of Kill Response

Bioassays of each isolate on second instars were performed as described in Section 2.3 except that five concentrations of 1 × 10^5^ to 1 × 10^9^ conidia/mL were used to inoculate larvae. Larvae mortality was evaluated at 24 h intervals over a period of 14 days. A concentration of 1 × 10^8^ conidia/mL was used to determine the LT_50_ value and the cumulative mortality after 14 days of incubation.

### 2.6. Statistical Analysis

Larval mortality and mycosed larvae caused by the 24 fungal isolates in the single-concentration bioassays were analyzed by one-way analysis of variance (ANOVA), with the Tukey HSD test (*p* < 0.05) to separate means, after checking for homogeneity of variances in accordance with the Levene test. Before the analysis, data were transformed to arcsine √x. Statistical tests were performed using R version 4.0 (https://www.r-project.org/, accessed on 1 March 2024). A correlation between larval mortality (based on the total number of insects treated) caused by the 24 fungal isolates and sporulation (based on the sporulation of dead insects) was performed by the Spearman Rank Correlation Coefficient test using SPSS ver. 21 for Windows.

The LC_50_ and LT_50_ values were calculated using Polo Plus© software version 1.0(LeOra Software, Berkely, CA, USA), and χ^2^ goodness-of-fit tests were performed for each isolate. Differences in LC_50_ and LT_50_ values among isolates were based on the non-overlap of 95% confidence intervals. The LT_50_ was calculated for the concentration needed to kill approximately 90% of the treated insects. We used Gehan–Breslow and Kaplan–Meier survival analysis and the non-parametric procedure LIFETEST to compare the effect of five isolates on *S. frugiperda* larval survival. A pairwise multi-comparison procedure (Long-Rank test, *p* < 0.05) was used to detect significant differences among treatments (SAS/STAT^®^, SAS Institute, Gary, NC, USA).

## 3. Results

### 3.1. Fungi Field Collection

**Figure 1:**
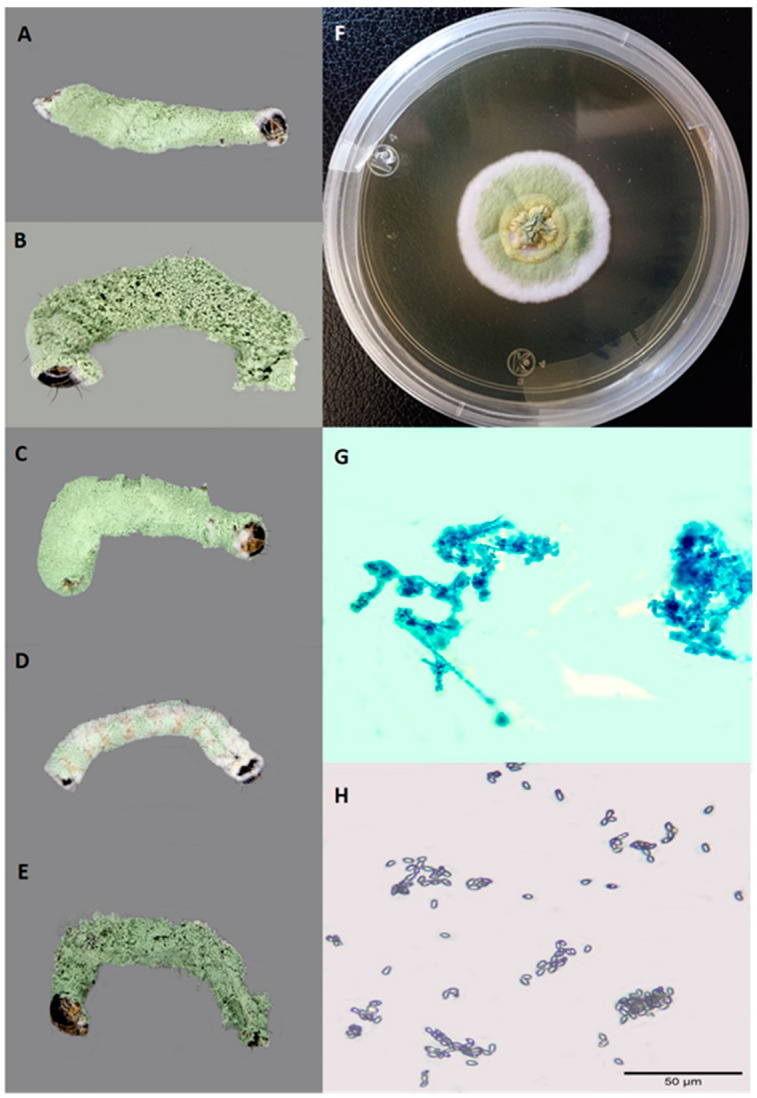
Morphological characteristics of *M. rileyi*. (**A**–**E**) Sporulation produced by isolates T9-21, Z30-21, PP48-21, J10-22, and L8-22 on *S. frugiperda* larvae, respectively, 8 days after inoculation. Whitish mycelium followed by light green spores can be observed. (**F**) *M. rileyi* colony on MPYA culture medium of 14 days after inoculation. A dense whitish cover is observed initially, which later turns into a light green colour. (**G**) Phialides in whorls and conidiophores. (**H**) Ovoid conidia dispersed and some of them form short chains.

Of a total of 295 collected larvae, 89 were infected by an entomopathogenic fungus. Based on the morphological characteristics, all isolates were identified as *M. rileyi*. Initially, these isolates formed a solid white covering on the MPLA culture medium and developed a light green coloration after ~9 days. When observed under the microscope, short and divergent chains of ovoid conidia produced on phyalides were observed (Figure 1).

In the first field collection (September 2021), the percentage of *S. frugiperda* larvae infected by *M. rileyi* was 23%, 30%, and 30% in El Trébol, Peña del Panal, and Zinapécuaro, respectively. In the second field collection (September 2022), the infection percentage was 48% and 90% for larvae collected at El Jacal and Lagunillas, respectively. Larvae with signs of infection by entomopathogenic viruses, bacteria, or other fungi species were not observed at any collection sites. Conidial germination of the 89 isolates mentioned above ranged from 3.3% to 98.5% (Table S1). From the total larvae collected, 1.96% (*n* = 5) were parasitized by tachinids (Diptera: Tachinidae).

### 3.2. Single-Concentration Bioassays

At a concentration of 1 × 10^8^ conidia/mL, significant differences were observed among isolates in the mortality of *S. frugiperda* second instars (F_23,72_ = 4.88, *p* < 0.0001) and the proportion of cadavers presenting sporulation (F_23,72_ = 6.87, *p* < 0.0001) at 13 days post-inoculation (Table 2). Mortality caused by isolate T1-21 (70%) was significantly lower than the remaining isolates, which caused mortality between 89% and 99%, and we did not observe significant differences among them.

Regarding sporulation, no significant differences were observed in most of the isolates (ranging from 85% to 98%) with the following exceptions: (i) the isolate T1-21 (60.5%) was significantly lower than the remaining isolates (ranging from 82 to 98%), with the exception of the isolate PP1-21 (80%); (ii) the isolate T69-21 (82%) was significantly lower than the isolate J10-22 (97%). A positive and significant correlation was observed 15 days post-inoculation between sporulation and host mortality (Spearman Rho = 0.55, *p* < 0.0001).

### 3.3. Genetic Characterization

**Figure 2:**
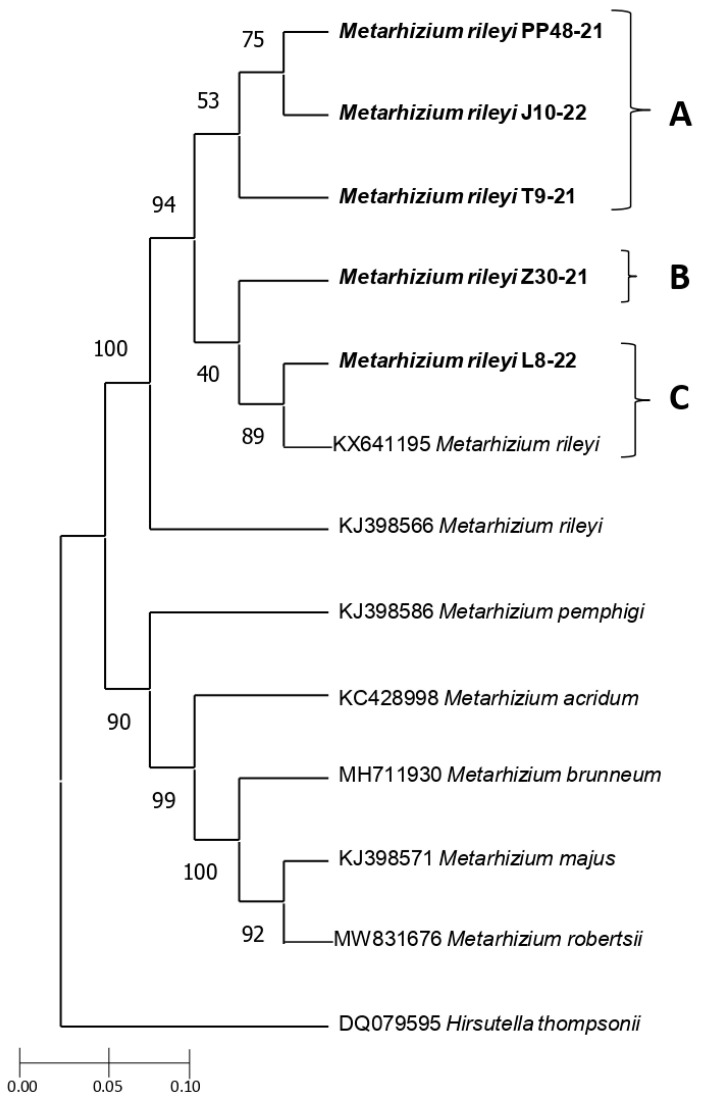
Phylogenetic tree inferred using the neighbor-joining method of 13 *β tubulin* sequences from entomopathogenic fungi isolates. Five sequences of *M. rileyi* (T9-21, Z30-21, PP48-21, J10-22, and L8-22 isolates) obtained from *S. frugiperda* larvae in five locations of the Michoacán state, Mexico, were clustered using the Tamura 3 model. Branch length is presented as the number of base substitutions per site. Three clades (A, B, and C) were grouped with *M. rileyi* sequences.

The five Mexican entomopathogenic fungi selected from different geographical points were grouped with *M. rileyi* sequences, based on their molecular structure using the *β-tubulin* gene sequences. The BLASTN analysis showed that the five sequences shared 99.32% similarity. In addition, when aligned for gaps, they showed a similarity of 99.08% and 98.47% with Gene bank sequences of *M. rileyi* (KX641195 and KJ398566, respectively). Phylogenetic analysis showed three strongly supported branches, one of them supported by *M. rileyi* isolates (Figure 2). The Mexican entomopathogenic fungi were grouped with 100% of branch support with the *M. rileyi* sequence reported by Kepler et al. [18]. The sequences of the five Mexican isolates were classified into three clades, denoted as A, B, and C. Isolates PP48-21, J10-22, and T9-21 showed a clade supported with 53% (clade A). In this clade, isolates PP48-21 and J10-22 were closely related (75%). The isolate Z30-21 (clade B) was modestly related (40%) to isolate L8-22 and one used as a reference (KX641195) (clade C). The isolates L8-22 and KX641195 were closely related (89%).

### 3.4. Bioassays with Five Selected Isolates

**Figure 3:**
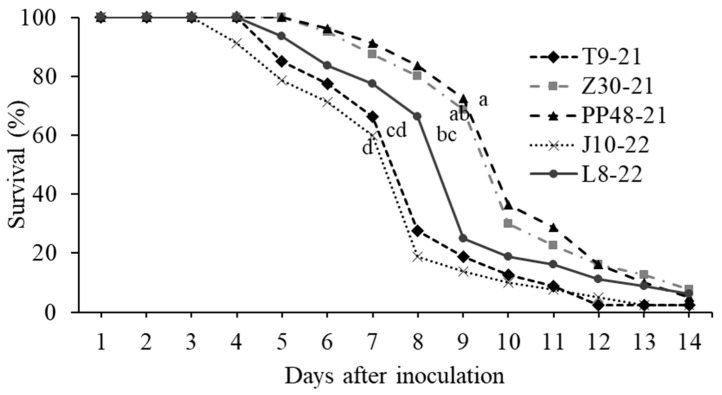
Gehan–Breslow and Kaplan–Meier survival curves of second-instar larvae of *S. frugiperda* after inoculation with a concentration of 1.0 × 10^8^ conidia/mL of five isolates of *M. rileyi*. Different letters indicate significant differences among isolates (log-rank test, *p* < 0.05).

The LC_50_ values for *S. frugiperda* second instars did not differ significantly among the five *M. rileyi* isolates, based on the overlap of 95% confidence intervals (Table 3). LC_50_ values ranged from 2.04 × 10^5^ to 1.05 × 10^6^ conidia/mL. At a concentration of 1.0 × 10^8^ conidia/mL, the T9-21, J10-22, and L8-22 isolates were the most virulent, with LT_50_ values of 7.40, 7.04, and 7.5 days, respectively, and no significant differences were observed among them (Table 3). Gehan–Breslow and Kaplan–Meier survival analysis revealed significant differences between *M. rileyi* isolates (log-rank test, χ^2^ = 76.04, *p* < 0.0001, Figure 2). The T9-21, J10-22, and L8-22 isolates caused lower survival than the remaining isolates. However, no significant differences were observed between the L8-22 isolate and PP48-21 (Figure 3).

## 4. Discussion

Several studies have reported that *S. frugiperda* larvae are naturally infected by *M. rileyi*. Therefore, this pathogen can be an important factor in the regulation of the populations of this pest [11,24,25,26,27,28]. A high incidence of epizootics caused by *M. rileyi* in maize crops has been reported, and the pathogen can infect different larval instars [24]. Variable percentages of natural infection by this fungus on *S. frugiperda* have been reported in several states of Mexico as follows: 8.6% in Chihuahua [41], 16% in Coahuila [42,43], 8.9% in Nayarit [44], 0.3–17% in Jalisco, 3.5–7.5% in Colima, 0.9–8% in Michoacán [45], and 3.05% in Chiapas [46]. In our study, the prevalence of *M. rileyi* infections of *S. frugiperda* in maize fields was higher across all collection sites (ranging from 23% to 90%) compared with the previous studies. However, our findings were consistent with natural infections observed outside of the Americas. For example, Visalakshi et al. [26] observed mycosis between 5.6% and 38% of *M. rileyi* on *S. frugiperda* larvae on maize and other crops in India. Ginting et al. [47] reported a 79% incidence of this fungus on this pest in Indonesia. In contrast, it was recently reported that fungal infection frequencies on *S. frugiperda* larvae on maize crops in China were very low (0.32%), with only two larvae infected, one by *B. bassiana* and another by *M. rileyi* [28]. This very low infection prevalence was attributed to the recent invasion of this insect in this country. In general, differences in the natural infection percentages may be due to different factors, including the number of collected larvae, incubation under laboratory conditions, and differences in the susceptibility of the insect host [42,48,49]. In addition, in field conditions, high humidity and high rainfall, as well as warm conditions, were very favorable for disease development in India [26].

Previous studies have shown a high prevalence and diversity of entomopathogenic fungi in Mexican *S. frugiperda* populations [41,42,43,44,45,50]. However, in our study, *M. rileyi* was the only entomopathogenic fungus species isolated from the larvae of this insect collected from maize crops along the sampled sites. Similarly, in other studies, this fungus was the most abundant or unique infecting *S. frugiperda* larvae (e.g., Michoacán, Colima, Jalisco, Tamaulipas, and Chiapas [45,46]. This may indicate the high adaptability and prevalence of *M. rileyi* on this host in specific environmental conditions. However, we did not discard the possibility of the presence of other entomopathogenic fungi established in maize fields in Michoacán state, as was reported by Lezama-Gutiérrez et al. [45] in *S. frugiperda* larvae and Huerta-Ramírez et al. [51] in soil samples.

Although most of the studies mentioned above did not include field environmental data, these have been performed during the rainy season, which coincides with the collection time of our study. However, in agreement with Fronza et al. [10], the scarcity of field studies, with detailed information about the adaptability of different *M. rileyi* isolates in a variety of environmental conditions, is a gap that must be filled.

One important step for the development of a fungal bioinsecticide resides in the selection of pathogenic and virulent candidate isolates [48]. In addition, the selection of a pathogen as a biocontrol agent requires that isolates be tested against an insect population from the locality in which the program is to be run [52]. In our study, 23 of the 24 *M. rileyi* isolates collected in the field and tested under laboratory conditions caused high rates of larval mortality (ranging from 80% to 98.7%), similar to studies performed on this pest in Colombia [24], India [26], and China [48,49].

In our study, a positive correlation between mortality and the proportion of cadavers presenting sporulation was observed 15 days post-inoculation. Similarly, Visalakshi et al. [26] observed that all *S. frugiperda* larvae that died due to an Indian *M. rileyi* isolate showed sporulation. However, in other host-entomopathogenic fungi systems, the proportion of cadavers presenting sporulation was low relative to the mortality host (e.g., *B. bassiana* vs. *Helicoverpa armigera* Hübner [53], and *B. bassiana* vs. *Diaphorina citri* Kuwayama [54]. The outgrowth and sporulation of entomopathogenic fungi on cadavers is a key source for the transmission of spores to susceptible hosts, which contributes to the overall performance of microbial control in crops [54].

Species of the genus *Metarhizium* evidenced an important genotypic variation, and even isolates of the same species show genetic variability, potentially related to their adaptation to different habitats [55,56]. *M. rileyi* has been extensively studied through the amplification of different genes, including elongation factor 1-α [27], ITS [49,57], and *β-tubulin* [18,29,58,59,60], among others. The *β-tubulin* gene can be used to determine evolutionary relationships among the sequences of Hypocreales [18,29] and basidiomycetous fungi [61], among others. In this study, phylogenetic analysis based on this gene showed that sequences of *M. rileyi* formed a distinct group within the different *Metarhizium* sequences analyzed. This is consistent with an in-depth study by Kepler et al. [18], where two *M. rileyi* sequences together formed a distinct group with respect to 56 *Metarhizium* sequences using *β-tubulin*, RPB1, RPB2, and TEF genes when a maximum likelihood phylogeny inferred from the analysis of a concatenated dataset was conducted. In the present study, the identification based on molecular characters by *β-tubulin* gene demonstrated that the five isolates corresponded to this species that was previously identified by morphological characteristics. The phylogenetic analysis showed that the five Mexican isolates were closely related (99% and 98%) to the isolate KX641195, which was obtained from *Spodoptera depravata* (Butler), and the isolate KJ398566, which was isolated from lepidopteran larvae in Brazil [18], respectively. In our study, three *M. rileyi* isolates (PP48-21, J10-22, and T9-21) formed a clade, suggesting that these isolates may have likely evolved from the same ecological niche, which coincides with their geographical proximity. In contrast, the isolates that were collected at greater geographical distances (L8-22 and Z30-21) were placed in two different clades. In this respect, future studies are needed to support these results using alternative protein-coding genes (e.g., RPB1, RPB2, and TEF, as described in Kepler et al. [18]) to resolve the phylogenetic relationships among Mexican *M. rileyi* isolates. Similarly, molecular methods such as amplification of elongation factor 1-α gene, simple sequence repeats (ISSR), and amplified fragment length polymorphism (AFLP) are also needed to determine *Metarhizium* species, genetic diversity, and population structure depending on their geographical distribution [62,63,64]. However, these authors determined that two *N. rileyi* isolates obtained from *S. frugiperda* larvae collected in the same location in Brazil were associated with different clades. In contrast, genetic analysis with ITS sequences showed that a Cuban *N. rileyi* isolate was closely related to an isolate collected in China. These genetic differences or similarities among *N. rileyi* isolates, independently of the region or the host from which they were isolated, were related to the high degree of polymorphism of this pathogen [62,63,65,66].

On the other hand, the pathogenicity of *M. rileyi* is a multi-faceted trait that depends on genetic and biological characteristics [60]. The genetic differences observed among the five *M. rileyi* isolates in this study did not always reflect differences in their pathogenicity since no significant differences were observed among their LC_50_ values. In contrast, isolates T9-21, J10-22, and L8-22 were the fastest to kill, causing lower survival rates. These results partially agree with those obtained by Pang et al. [49], who reported that the pathogenicity and speed to kill of two genetically similar isolates of *M. riley* (XSBN200920 and HNQLZ200714), collected from *S. frugiperda* larvae on maize in different locations in China, varied significantly in third and fifth instar *S. frugierda* larvae. They also related the biological activity of the most pathogenic isolate (XSBN200920) to its potent antioxidant potential and the highest growth rate. Further studies are needed to identify the factors associated with the insecticide activity of the Mexican *M. rileyi* isolates studied here, particularly those related to the speed of kill.

Some of the LC_50_ values obtained in the present study were similar to those determined in other studies performed in Colombia using the spraying method onto plants infested with second instars of *S. frugiperda* (ranging from 9.8 × 10^3^ to 2.2 × 10^5^ conidia/mL) [24]. A concentration of 1 × 10^6^ or 1 × 10^7^ conidia/mL of two *M. rileyi* isolates from China caused 50% mortality against the third instar of the same insect species we studied [49]. Similarly, LT_50_ values of 5–7 days [24] and 12 days [48] for isolates from Colombia and China, respectively, were reported for the *S. frugiperda* second instars. In general, several factors can influence the susceptibility of insects to infection by entomopathogenic Hyphomycetes [67], including developmental stages of the host [53], cuticle sclerotization [68], and insect immune system [27]. Additionally, the expression of a diversity of enzymes is crucial in the penetration process of the fungi, such as proteases, chitinases, actins, and hydrophobins, as well as genes such as *mad1* and *mad2* that code for adhesins. These genes are related to the host infection process [69]. In this regard, the expression of antioxidant stress genes, such as SOD and CAT, and antioxidant enzymes were identified as the key factors responsible for the variation in virulence between two *M. rileyi* isolates collected in China [49]. Cruz-Avalos et al. [70] also identified the virulence genes *mad1* and *mad2* in *M. anisopliae*, as well the genes *hyd*1 and *hyd*2, in *B. bassiana* isolated from soil samples of maize-growing fields. However, no correlation was observed between the presence of virulence genes and biological activity. Although a high insecticide activity was detected in the five Mexican *M. rileyi* isolates studied here, further studies are needed to identify the factors associated with this biological activity and to identify which of these factors are the most important during the infection process, particularly those related to the speed of kill.

## 5. Conclusions

Our study provides valuable insights into the phylogeny and pathogenicity of five Mexican isolates of *M. rileyi* on the second instars of *S. frugiperda*. The Mexican isolates were morphologically identified as *M. rileyi* and genetically related to other isolates of this fungal species. Although all Mexican isolates exhibited similar pathogenicity, the T9-21, J10-22, and L8-22 isolates emerged as the most promising candidates as potential biological insecticides because these were the most virulent isolates. Further studies are needed to determine the genetic diversity of these isolates and their virulence factors.

## Figures and Tables

**Table 1:** Locations and date of collection of *M. rileyi* isolated from *S. frugiperda* larvae.

Collection Site/Municipality	Collection Date	Number of Larvae Collected	Coordinates
El Trébol, Tarímbaro	2 September 2021	137	19°46′12.5076″ N—101°09′17.6724″ W
Peña del Panal, Tarímbaro	7 September 2021	78	19°46′27.8976″ N—101°11′04.452″ W
Zinapécuaro, Zinapécuaro	15 September 2021	44	19°50′46.2703″ N—101°01′40.2930″ W
Lagunillas, Pátzcuaro	7 September 2022	25	19°36′40.554″ N—101°25′23.7″ W
El Jacal, Chucándiro	21 September 2022	11	19°53′16.3644″ N—101°20′12.1632″ W

**Table 2:** Mortality and sporulation caused by a concentration of 1 × 10^8^ conidia/mL of 24 *M. rileyi* isolates on second instar *S. frugiperda* larvae.

Isolates	Mortality (%) ± SEM	Sporulation (%) ± SEM
T1-21	70.0 ± 4.1 a	60.5 ± 1.7 a
T69-21	90.0 ± 2.0 b	82.0 ± 1.0 bcd
T70-21	90.0 ± 3.0 b	84.9 ± 5.3 bcdef
T72-21	95.0 ± 2.0 b	88.0 ± 3.6 bcdef
T5-21	93.7 ± 2.4 b	87.6 ± 4.7 bcdef
**T9-21**	97.5 ± 3.2 b	94.6 ± 2.3 bcdef
T8-21	95.0 ± 3.5 b	81.3 ± 2.2 bc
T2-21	97.5 ± 1.4 b	91.1 ± 1.3 bcdef
PP1-21	93.7 ± 3.7 b	80.2 ± 3.4 ab
PP12-21	95.0 ± 2.0 b	81.5 ± 1.8 bc
PP18-21	88.7 ± 4.3 b	88.4 ± 4.5 bcdef
**PP48-21**	97.5 ± 1.4 b	94.9 ± 3.0 bcdef
Z2-21	93.7 ± 1.2 b	84.0 ± 2.1 bcdef
**Z30-21**	97.5 ± 1.4 b	96.1 ± 2.5 cdef
Z32-21	95.0 ± 2.0 b	88.0 ± 2.7 bcdef
Z33-21	90.0 ± 2.0 b	93.3 ± 3.4 bcdef
J5-22	96.2 ± 2.4 b	94.7 ± 2.1 bcdef
J6-22	93.7 ± 1.2 b	92.0 ± 1.5 bcdef
J9-22	92.5 ± 3.2 b	90.5 ± 2.7 bcdef
**J10-22**	97.5 ± 1.4 b	97.4 ± 1.5 ef
**L8-22**	98.7 ± 1.2 b	98.7 ± 1.2 bcdef
L9-22	96.2 ± 1.2 b	90.8 ± 2.5 bcdef
L11-22	97.5 ± 1.4 b	96.2 ± 1.3 def
L12-22	92.5 ± 1.4 b	89.1 ± 2.3 bcdef

In bold, the five isolates selected to determine their phylogenetic relationships using the *β-tubulin* gene analysis and to compare their biological activity in terms of median lethal concentration, median lethal time, and larval survival. Data represent means ± SEM. Within the same column, data followed by the same letter are not significantly different (Tukey HSD mean separation; *p* > 0.05).

**Table 3:** Median lethal concentrations and median lethal times of *M. rileyi* on second instar *S. frugiperda* larvae inoculated with five different isolates.

Isolates	Days Post- Inoculation	Slope ± SEM	LC_50_ (Conidia/mL)	Lower-Upper Limits	χ^2 a^	LT_50_ (Days Post-Inoculation)	Lower-Upper Limits
T9-21	13	0.61 ± 0.07	1.05 × 10^6^	1.9 × 10^5^–3.49 × 10^6^	4.05	7.40	7.01–7.76
Z30-21	13	0.41 ± 0.05	2.15 × 10^5^	5.9 × 10^4^–7.3 × 10^5^	3.94	9.46	9.04–9.90
PP48-21	13	0.48 ± 0.06	4.18 × 10^5^	1.3 × 10^5^–2.7 × 10^6^	4.87	9.65	9.30–10.0
J10-22	12	0.56 ± 0.06	2.04 × 10^5^	7.7 × 10^4^–4.2 × 10^5^	2.20	7.04	6.59–7.47
L8-22	10	0.39 ± 0.05	7.85 × 10^5^	2.5 × 10^5^–1.8 × 10^6^	2.36	7.49	7.04–7.92

Analyses were performed using the PoloPlus program version 1.0. ^a^ Goodness-of-fit probit model based on three degrees free.

## Data Availability

The sequences were submitted to the GenBank public collection of the National Center for Biotechnology Information (NCBI). The accession numbers are shown in Section 2.4.

## Supplementary Materials

The following supporting information can be downloaded at: https://www.mdpi.com/article/10.3390/jof10060416/s1. Table S1. Conidial germination of 89 isolates collected from maize plants and morphologically identified as *M. rileyi*.
