# Supplementary material for: Natural Prevalence, Molecular Characteristics, and Biological Activity of Metarhizium rileyi (Farlow) Isolated from Spodoptera frugiperda (J. E. Smith) Larvae in Mexico
Source: J Fungi (Basel). 2024 Jun 8;10(6):416. doi: 10.3390/jof10060416 (PMC11204773; doi:10.3390/jof10060416)
Supplement: Supplementary file 1 [file jof-10-00416-s001.zip › jof-2974604-supplementary.pdf]

Table S1. Conidial germination of 89 isolates collected from maize plants and morphologically identified as *M. rileyi*.

Isolates	Conidial germination (% ± SE)
J1-22	71.3 ±1.3
**J10-22**	**98.5 ± 0.3**
J11-22	67.8 ± 2.7
J2-22	44.0 ± 3.7
J3-22	8.0 ± 2.7
J4-22	61.5 ± 1.6
**J5-22**	**98.0 ± 0.4**
**J6-22**	**95.5 ± 0.3**
J7-22	82.3 ± 1.3
**J9-22**	**96.5 ± 0.3**
**L11-22**	**97.3 ± 0.5**
**L12-22**	**96.0 ± 0.4**
L18-22	65.5 ± 2.1
L20-22	61.0 ± 3.7
L21-22	86.3 ± 2.3
L22-22	80.3 ± 1.7
L23-22	69.8 ±2.7
L24-22	77.8 ± 3.1
L25-22	49.3 ± 1.7
L4-22	81.8 ± 1.2
**L8-22**	**98.5 ± 0.3**
**L9-22**	**97.3 ± 0.3**
P70-21	15.3 ± 3.7
P73-21	2.5 ± 1.4
P74-21	75.8 ±5.3
P75-21	68.8 ± 1.3
P77-21	3.3 ± 2.6
P78-21	50.0 ± 1.2
**PP1-21**	**97.3 ± 1.1**
**PP12-21**	**97.0 ± 0.4**
PP15-21	36.3 ± 2.4
**PP18-21**	**97.3 ± 0.6**
PP23-21	68.3 ± 1.4
PP29-21	51.8 ±1.2
PP35-21	60.8 ± 2.7
PP40-21	80.0 ± 2.0
PP42-21	73.8 ± 2.4
**PP48-21**	**98.0 ± 0.4**
PP5-21	82.0 ± 1.1
PP53-21	30.0 ± 2.0
PP56-21	65.8 ± 3.3
PP60-21	66.3 ± 6.3

PP63-21	14.5 ± 5.4
PP66-21	13.8 ± 8.0
PP7-21	74.3± 1.9
**T1-21**	**96.0± 0.4**
T110-21	45.0± 2.0
T119-21	81.3 ± 1.3
T12-21	54.3 ± 7.1
T15-21	29.3 ± 3.9
T19-21	79.3± 1.5
**T2-21**	**97.0± 0.4**
T22-21	49.0± 3.3
T23-21	77.0± 1.8
T25-21	34.3 ± 1.5
T28-21	75.8 ± 3.4
T31-21	78.8 ±1.3
T35-21	60.8 ±2.2
T42-21	37.0±2.1
T46-21	49.5± 4.2
T48-21	82.5± 1.4
**T5-21**	**95.8± 0.3**
T50-21	51.8 ± 4.3
T52-21	80.5 ± 2.1
T6-21	80.8 ± 2.5
T60-21	58.8 ± 3.1
T64-21	82.5±1.4
**T69-21**	**95.8± 0.5**
**T70-21**	**96.5± 0.3**
T71-21	44.5 ± 2.1
**T72-21**	**98.3 ± 0.8**
**T8-21**	**96.5 ± 0.6**
T80-21	80.0 ±2.0
T88-21	38.8 ± 1.3
**T9-21**	98.5 ± 0.3
T95-21	66.3 ± 2.4
Z10-21	66.8 ± 2.0
Z14-21	48.0 ± 2.0
Z18-21	61.0 ±6.7
**Z2-21**	96.8 ±0.8
Z21-21	76.3 ± 2.4
Z26-21	8.0 ± 4.9
**Z30-21**	**98.0 ± 0.4**
**Z32-21**	**96.8 ± 0.8**
**Z33-21**	**96.5 ± 0.3**
Z37-21	4.5 ± 2.6
Z40-21	5.0 ± 1.8
Z41-21	62.0 ± 5.8

Z43-21	71.8 ± 4.2

In bold, the 24 isolates that were evaluated on *S. frugiperda* second instars at a concentration of 1.0 × 10^8 conidia/mL
